# TB Screening in Canadian Health Care Workers Using Interferon-Gamma Release Assays

**DOI:** 10.1371/journal.pone.0043014

**Published:** 2012-08-20

**Authors:** Alice Zwerling, Mihaela Cojocariu, Fiona McIntosh, Filomena Pietrangelo, Marcel A. Behr, Kevin Schwartzman, Andrea Benedetti, Nandini Dendukuri, Dick Menzies, Madhukar Pai

**Affiliations:** 1 Department of Epidemiology, Biostatistics & Occupational Health, McGill University, Montreal, Canada; 2 Respiratory Epidemiology & Clinical Research Unit, Montreal Chest Institute, Montreal, Canada; 3 Department of Medicine, McGill University Health Centre, Montreal, Canada; 4 Department of Occupational Health & Safety, McGill University Health Center, Montreal, Canada; 5 Research Institute of the McGill University Health Center, Montreal, Canada; The University of Melbourne & The Murdoch Childrens Research Institute & The Royal Children's Hospital Melbourne, Australia

## Abstract

**Background:**

While many North American healthcare institutions are switching from Tuberculin Skin Test (TST) to Interferon-gamma release assays (IGRAs), there is relatively limited data on association between occupational tuberculosis (TB) risk factors and test positivity and/or patterns of test discordance.

**Methods:**

We recruited a cohort of Canadian health care workers (HCWs) in Montreal, and performed both TST and QuantiFERON-TB Gold In Tube (QFT) tests, and assessed risk factors and occupational exposure.

**Results:**

In a cross-sectional analysis of baseline results, the prevalence of TST positivity using the 10 mm cut-off was 5.7% (22/388, 95%CI: 3.6–8.5%), while QFT positivity was 6.2% (24/388, 95%CI: 4–9.1%). Overall agreement between the tests was poor (kappa = 0.26), and 8.3% of HCWs had discordant test results, most frequently TST−/QFT+ (17/388, 4.4%). TST positivity was associated with total years worked in health care, non-occupational exposure to TB and BCG vaccination received after infancy or on multiple occasions. QFT positivity was associated with having worked as a HCW in a foreign country.

**Conclusions:**

Our results suggest that LTBI prevalence as measured by either the TST or the QFT is low in this HCW population. Of concern is the high frequency of unexplainable test discordance, namely: TST−/QFT+ subjects, and the lack of any association between QFT positivity and clear-cut recent TB exposure. If these discordant results are indeed false positives, the use of QFT in lieu of TST in low TB incidence settings could result in overtreatment of uninfected individuals.

## Introduction

Fuelled by the HIV epidemic, the emergence of multi- and extensively drug resistant tuberculosis (MDR & XDR-TB) and an increasingly mobile population, tuberculosis (TB) continues to be a global concern and a serious risk to health care workers (HCW) worldwide [1], [2], [3], [4], [5]. In many high income countries, HCWs are screened at regular intervals for Latent TB Infection (LTBI) in an effort to identify new infections that can be targeted for preventive therapy [6], [7], [8].

Traditionally TB screening in HCWs has been conducted using the Tuberculin Skin Test (TST), a test with known limitations [9], [10], [11]. Recently, Interferon gamma release assays (IGRAs) are being increasingly used for LTBI screening. Several systematic reviews have suggested that IGRAs are as sensitive, and more specific than the TST in identifying LTBI, particularly in low TB incidence settings [12], [13], [14], [15]. However, the use of IGRAs for routine screening of HCWs remains an area of controversy, and the Canadian guidelines among others have not endorsed IGRAs for serial testing in this population [16], [17]. Despite this, the use of IGRAs in routine screening of HCWs is increasing, and with it, a large number of studies have reported high within subject variability, high rates of discordant test results and high rates of conversions and reversions among HCWs screened with these novel assays [18], [19].

We recruited HCWs from a low TB incidence setting into a prospective longitudinal cohort, in which HCWs are undergoing annual testing with TST and QFT. At intake into this cohort all participants underwent baseline testing. Primary objectives of this analysis were a) to assess the association between test positivity and occupational TB exposure in a cohort of Canadian HCWs; and b) to assess the association between patterns of test discordance and occupational TB exposure and other TB risk factors.

## Materials and Methods

### Study Setting & Population

This study was conducted at the McGill University Health Centre (MUHC), a university affiliated hospital network that employs over 11,500 health care and other personnel at seven hospitals in Montreal. TB incidence is low in Quebec with an annual incidence rate of 2.5 per 100,000 persons. Over the past decade, approximately 100–150 active TB cases have been reported annually on the island of Montreal, an incidence rate of approximately 7.1/100 000 persons per year [20].

HCWs at the MUHC undergo TB screening both as part of pre-employment health evaluations and through regular occupational health and safety monitoring. The majority of employees are to be screened for LTBI on an annual schedule, while those in high risk settings such as mycobacteriology laboratories are screened every 6 months. TB screening of MUHC HCWs has been traditionally done using the TST and is conducted by the Department of Occupational Health and Safety (OHS).

### Study Design & Ethics approval

HCWs were approached for participation in the study at their TB screening visit with the OHS. In order to be eligible for the study, participants must have been due for scheduled TB screening, and could not have had a positive TST (≥10 mm induration) in the past. Therefore, ‘baseline testing’ refers to the first measurement for this study; for some participants this was their first ever TST, while others had been undergoing annual occupational TB screening, with negative test results in the past. HCWs who provided written informed consent were recruited to our prospective longitudinal cohort, and are now being followed and tested annually with both the TST and QFT.

At the baseline study visit, the blood draw was first performed for the QFT test, and subsequently the TST was placed. Finally, HCWs completed an interviewer-facilitated questionnaire covering occupational and non-occupational TB exposure. The study was approved by the ethics review board of the MUHC.

### Tuberculin Skin Test

The TST was performed using the Mantoux method by a trained nurse hired by the research study; 5TU (0.1) ml of Tubersol PPD (Aventis Pasteur) was used, and results read as per the Canadian TB Standards [8]. In cases where the HCW had not been tested with the TST within the last 10 years, a 2 step TST was performed if the initial result was negative (<10 mm). Upon initial testing, an induration of ≥10 mm was considered positive [6], [8].

### Interferon-gamma release assay testing

The QuantiFERON-TB Gold In-Tube test (QFT) (Cellestis Ltd/Qiagen, Carnegie, Australia) was performed as per the manufacturer's instructions in a research lab at the Montreal General Hospital. The QFT result was considered positive if the interferon-gamma response in the TB antigen tube minus the response in the nil tube was ≥0.35 IU/ml as per the manufacturer's guidelines. Quantitative values of IFN-gamma were also recorded; as per manufacturer's suggestions all values greater than 10 were truncated at 10.

### Ascertainment of TB Exposure

HCWs completed a questionnaire covering demographics, travel history, training and work history in health care, health care work in foreign countries, potential non-occupational exposure to TB, and potential exposure to TB in the occupational environment. Known occupational exposures are charted in the personnel files maintained by the Department of Occupational Health and Safety.

While there is no gold standard for LTBI, we hypothesized that a higher level of exposure to TB correlates with a higher probability of infection. We identified key occupational exposures based on *a priori* risk factors including: a) unprotected exposure to a smear positive infectious TB patient, b) direct contact with TB patients, c) type of HCW, and d) years worked in health care [18].

### Management of Positive Results

As per the current MUHC policy, participants with a positive TST result were referred for chest x-ray plus clinical evaluation by a pulmonologist. When judged appropriate, LTBI treatment was recommended by the pulmonologist. The same process applied to participants with a positive QFT result, regardless of the accompanying TST result.

### Statistical Analysis

All analyses were performed with Stata, version 11 (Stata Corp, Texas, USA). Percent agreement and kappa statistics, were calculated to estimate test concordance. To assess the association between test positivity (separately for TST and QFT) and the occupational exposure variables of interest, we performed multivariable logistic regression. Variable selection for the model was done using *a priori* clinical knowledge. The association between risk factors and the joint patterns of TST and QFT results was assessed by nominal multinomial logistic regression. Four patterns were assessed: concordant negatives (base or reference outcome), concordant positives, discordant QFT+/TST− and discordant QFT−/TST+. Odds ratios and their accompanying 95% confidence intervals were estimated from the multivariable polytomous logistic regression model.

## Results

From May 2007 to December 2011, we approached 638 HCWs who had been referred for TST. Three hundred and ninety seven HCWs (62%) provided informed consent, completed the questionnaire, and underwent study testing procedures. Most common reasons for non-participation were lack of time, dislike of needles and/or of blood draw. Incomplete and invalid tests were excluded (including tests which could not be performed due to processing or laboratory errors. Completed questionnaires, and valid test results were available for 388 HCWs.

Among the 388 study participants with complete results, 87.9% (341/388) had had a prior TST planted either through routine TB screening at the MUHC or at another location prior to employment at the MUHC (but not within the preceding 3 months), 41/388 (11.3%) had never had TST performed previously while 6/388 (1.6%) could not recall. A second step TST was performed on 41 TST naïve HCWS and 59 HCWs who had not been tested with TST within the last ten years. Among those previously tested, 332/341(97.4%) had documented TST negative while 9/341 (2.6%) had been previously tested with TST but had never been read.

### Participant Demographics & TB Exposure

Participant characteristics are displayed in Table 1. The majority of HCWs were female (288/388, 74%) and the median age was 34.4 years (Inter-quartile range, IQR: 26.9–44.7 years). The majority were Canadian-born non-aboriginal (272/388 70%), and the majority of foreign born participants (86/114, 75%) had arrived in Canada more than 5 years prior to study enrolment. HCWs had worked a median of 5 years (IQR 2- 12 years) in the health care services sector. Prior BCG vaccination was reported by 36.1% (140/388) of the cohort.

**Table 1:** Participant Characteristics (N = 388).

Participant Characteristics		N (%)
Age	Median	34.4 yrs
	IQR	26.9–44.7 yrs
Sex	Female	288 (74.2%)
	Male	100 (25.8%)
Country of birth	Canadian born (non-aboriginal)	272 (70.1%)
	Canadian born (aboriginal)	2 (0.5%)
	Foreign born: low TB inc. (≤25/100, 000)	63 (16.3%)
	Foreign born : moderate TB inc (26–100/100,000)	23 (5.9%)
	Foreign born: high TB inc (>100/100,000)	28 (7.2%)
Educational level	High school or less	46 (11.9%)
	College or university degree	231 (59.5%)
	Post graduate degree	108 (27.8%)
	Did not report	3 (0.8%)
Job category	Non- clinical staff	120 (30.9%)
	Nursing staff	94 (24.2%)
	Medical doctors	40 (10.3%)
	Other clinical staff	115 (29.6%)
	Laboratory staff	19 (4.9%)
BCG vaccination	No vaccination	248 (63.9%)
	At birth or within 1 year	57 (14.7%)
	Post infancy	36 (9.3%)
	Received multiple BCG vaccinations	7 (1.8%)
	Unknown timing	40 (10.3%)
Direct contact with a patient with TB	Yes	145 (37.4%)
	No	243 (62.6%)
Non-occupational TB exposure*		
TB case in same household		9 (2.3%)
TB case at school		6 (1.6%)
TB case in same room		15 (3.9%)
Friends with TB case		10 (2.6%)
No TB exposure in the community		361 (93%)
Total years worked in health care	Median	5 yrs
	IQR	(IQR: 2–12 yrs)
Worked as a HCW outside of Canada?	No	343 (88.4%)
	Yes	45 (11.6%)
Travel outside of Canada (>1 month)	No	279 (71.9%)
	Yes	109 (28.1%)
Prior TST	Yes – negative	332 (85.6%)
	Yes - but not read	9 (2.3%)
	No	41 (10.6%)
	Don't know	6 (1.6%)

* Not mutually exclusive categories, therefore will not total to 100.

The median number of years since the last TST prior to study recruitment was 2.2 years, (IQR: 1.4–3.6 yrs) and the median number of TST tests performed prior to study enrollment was 2, (IQR:1–3) with some OHS charts reporting up to 14 TST tests per HCW.

### Baseline test positivity

The prevalence of TST positivity using the 10 mm cut-off was estimated to be 5.7% (22/388, 95%CI: 3.6–8.5%), while QFT positivity was 6.2% (24/388, 95%CI: 4–9.1%), with only one indeterminate QFT result. Quantitative TST results ranged from 0 to 25 mm. When appropriate, two step TST was performed, and in 5/100 (5%) cases HCWs became TST positive upon second step testing, these HCWs were referred for follow-up but were not considered positive for subsequent analyses. QFT results (TB Ag- nil) ranged from 0–10 IU/ml. All HCWs with positive TST results were referred to see a pulmonologist, however in 7 cases, HCWs did not make a follow-up appointment. Treatment was recommended in 7/15 (46.7%) HCWs; 5 completed therapy, while 2 refused treatment. Treatment was not recommended in the remaining 8 HCWs, either due to concerns with drug interactions (n = 1), age over 35 years (n = 2) or because they were considered false-positives (n = 5).

Positivity estimates stratified by prior TST status, test type, BCG vaccination status, and country of birth are displayed in Table 2. Among those with no prior TST history, the QFT positivity rate was 4.9% (2/41, 95%CI: 0.6–16.5%) while the TST rate was almost twice that at 9.8% (4/41, 95%CI: 2.7–21.9%). When restricted to those with prior negative TST the test positivity rates for both tests decreased and the rate for QFT positivity became almost 2 percentage points higher than that of TST. Among BCG vaccinated HCWs, test positivity was slightly lower for QFT than TST.

**Table 2:** Test Positivity among HCWs, stratified by test type, prior TST status, BCG vaccination status and country of origin.

Subgroup	TST Positive/Tested (%)	95%CI	QFT Positive/Tested (%)	95%CI
**Overall**	22/388 (5.7%)	3.6–8.5%	24/388 (6.2%)	4.0–9.1%
BCG Vaccinated	18/140 (12.9%)	4.6–1.0%	13/140 (9.3%)	5.0–15.4%
No history of BCG	4/248 (1.6%)	7.8–19.6%	11/248(4.4%)	2.2–7.8%
Canadian Born**	5/274 (1.8%)	0.4–4.1%	11/274 (4.0%)	2.0–7.1%
Foreign Born	17/114 (14.9%)	0.6–4.2%	13/114 (11.4%)	6.2–18.7%
Foreign Born (High TB Incidence)	4/28 (14.3%)	8.9–22.8%	3/28 (10.7%)	2.3–28.2%
Prior TST	4/41 (9.8%)	2.7–21.9%	2/41 (4.9%)	0.6–16.5%
No prior TST Unknown prior TST	5/15 (33.3%)	11.8–61.6%	3/15 (20.0%)	4.2–48.1%
Prior Negative TST	13/332 (3.9%)	2.1–6.6%	19/332 (5.7%)	3.5–8.8%

** Including Canadian-born aboriginals.

Note: 2 step TST: 5/100, (5.0%).

### Test agreement

**Figure 1:**
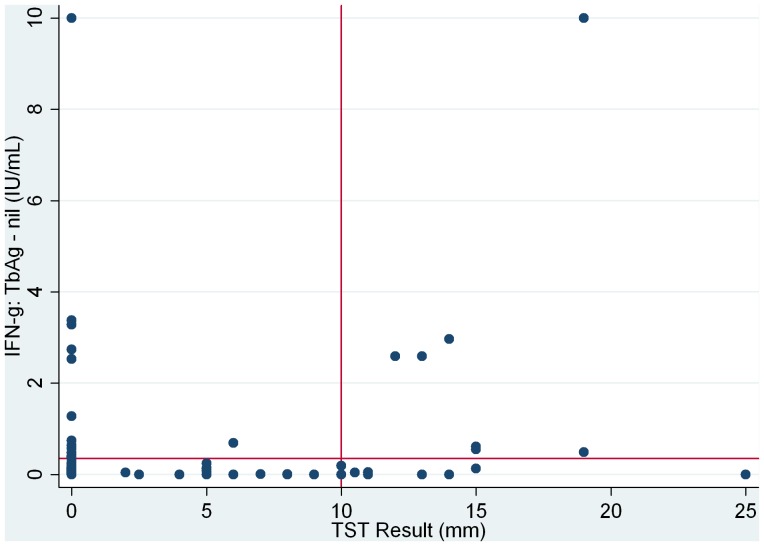
Scatterplot of quantitative TST and QFT results (n = 388)*. *2 points not visible on graph: a)TST = 0, IFN-g = 10 & b)TST = 17, IFN-g = 10.

Concordance between quantitative TST and QFT results is displayed in Figure 1. Three hundred and forty-eight (89.7%) HCWs were found to be negative by both tests, while 7 (1.8%) were concordant positives (lower left and upper right hand quadrants of Figure 1). TST positive/QFT negative discordant results were found in 15/388 cases (3.9%) and TST negative/QFT positive in 17/388 cases (4.4%).

Among those with no history of BCG vaccination, only 3/248 (1.2%) were TST+/QFT− discordant, while 10/248 (4%) were TST−/QFT+ discordant. Among the BCG vaccinated, a much higher rate of TST+/QFT− discordance was observed (12/140 ,8.6%), compared to TST−/QFT+ discordance (7/140 ,5%). Overall agreement between the tests was 91.7% with a kappa value of 0.26 which can be interpreted as fair to poor agreement [21], [22]. When the analysis was restricted to only those HCWs with BCG vaccination, the agreement decreased slightly to 86.3%, while the kappa value improved slightly to 0.3.

As seen in Figure 1, only 4 points (1 not shown) suggest clearly concordant positive results, while the remaining points (although still concordantly positive are close to the QFT positivity cut-off both among concordant positives and discordant TST+/QFT−. There was no statistically significant difference between the median IFN-gamma value among concordant positives (median:2.6, IQR:0.55–2.97) and among the discordant QFT +/TST negatives (median: 0.69, IQR:0.47–2.74).

When we considered a more stringent cut-off based on visual inspection of the scatterplot, we found that by employing a cut-off of 1.0 IU/ml for the QFT test , 13 HCWs would be reclassified as QFT negative; among these, 3/13 (23%) were TST positive leading to a lower QFT positivity rate of 11/388 (2.8%). This reclassification would lead to a TST+/QFT− discordance rate of 4.6% while the TST−/QFT+ discordance rate would decrease to 1.8%.

### Multivariable logistic regression

The final multivariable model included the following independent variables: age, sex, non-occupational TB exposure (yes/no), birth in a country with high TB incidence, type of health care work (nurse, doctor, clinical staff, lab staff or nonclinical support staff), Total years worked and or spent training in health care, Travel more than one month outside Canada, worked as aHCW in a foreign country, BCG vaccination at birth or unknown timing, BCG vaccination post-infancy or multiple BCG vaccinations, self-reported exposure to active TB patients, documented occupational exposure to TB, age at immigration, birth outside of Canada, and finally prior negative TST (Table 3).

**Table 3:** Multivariable logistic regression model assessing association between test positivity and risk factors.

Risk Factors		Association with TST positivity		Association with QFT positivity	
		adj. OR	95% CI	adj. OR	95% CI
Sex	Female	1			
	Male	2	(0.5–7.9)	11.4	(0.5–3.9)
Age (yrs)		0.98	(0.9–1.1)	1.03	(0.98–1.1)
Birth in a low/moderate TB incidence country		1		1	
Birth in a country with high TB incidence		2.31	(0.3–16.7)	1.57	(0.3–7.6)
Job Category	Non-clinical staff	1		1	
	Doctors	4.18	(0.4–45.6)	1.9	(0.4–10.1)
	Nurses	1.09	(0.2–7.9)	0.7	(0.2–3.2)
	Clinical staff	1.88	(0.3–10.8)	0.78	(0.2–2.9)
	Laboratory staff	4.48	(0.45–44.7)	-	-
Non-Occupational TB Exposure					
No reported exposure		1		1	
Self-reported TB exposure		**11.7**	**(2.5–55.7)**	0.45	(0.05–3.9)
Total years worked in health care (yrs)		**1.09**	**(1.003–1.2)**	0.99	(0.9–1.1)
Travel outside Canada >1 month	No	1			
	Yes	2.31	(0.7–8.2)	11.13	(0.4–3.1)
Worked as a HCW in a foreign country	No	1		**1**	
	Yes	1.09	(0.2–5.9)	**6.44**	**(1.6–25.0)**
Self-reported occupational contact with a patient with active TB	No	1		1	
	Yes	0.88	(0.54–1.44)	0.94	(0.6–1.4)
Known unprotected occupational exposure	No	1		1	
	Yes	1.35	(0.91–2.0)	0.95	(0.7–1.3)
BCG Vaccination					
No Vaccination		1		1	
Vaccination at birth/timing unknown		4.17	(0.81–21.49)	0.97	(0.3–3.2)
Vaccination post-infancy or >1 BCG		**23.05**	**(3.9–135.2)**	2.7	(0.8–9.5)
Country of Birth	Canada	1		1	
	Foreign-born	1.42	(0.2–8.9)	2.06	(0.5–9)
Age at immigration to Canada (yrs)		1.06	(0.99–1.1)	0.96	(0.9–1.0)
Prior TST	No prior TST or not read	1		1	
	Prior negative TST	**0.065**	**(0.01–0.3)**	0.54	(0.1–2.0)

Statistically significant results are presented in bolded text.

In the final model, non-occupational exposure to TB (OR = 11.7, 95%CI: 2.5–55.7), total years worked in health care (OR = 1.09 for every additional year, 95%CI: 1.003–1.2), BCG vaccination after infancy and or multiple BCG vaccinations (OR = 23.05, 95%CI:3.9–135.2) were all statistically significantly associated with TST positivity. Prior negative TST (OR = 0.065, 95%CI: 0.01–0.3) had a strong inverse association with TST positivity in the present study. The only variable to be statistically significantly associated with QFT positivity was having worked as a HCW in a foreign country (OR = 6.44, 95%CI: 1.6–25.0), (Table 3).

We performed a stratified analysis of only those HCWs with previous negative TST. Among this group, we found the same significant variables associated with TST positivity in the multivariable regression with the exception of total years worked in health care was no longer significant in the model. This suggests that the cumulative risk of years working in health care is not a significant predictor among those who have been routinely screened in this context.

### Patterns of discordance and their association with TB risk factors

Using a multinomial logistic regression model we assessed the potential association between risk factors and 4 patterns of test concordance/discordance: concordant negatives, concordant positives, and two types of discordance: QFT+/TST− and QFT−/TST+. All outcomes were compared to the base outcome of concordant negatives (Table 4). In multivariable analysis the only three variables significantly associated with concordant positive results were BCG vaccination after birth and/or multiple vaccinations (OR = 36.32, 95%CI: 1.73–761.31), having worked in a foreign country as a HCW (OR = 30, 95%CI: 1.34–669.8), and prior negative TST, which showed a statistically significant protective effect (OR = 0.02 95%CI: 0.001–0.57)

**Table 4:** Multinomial logistic regression model, assessing the relationship between risk factors and patterns of concordant and discordant test results.

		Concordant Negatives (Reference)	Odds Ratios (95%CI)		
			Concordant Positives	Discordant TST+/QFT−	Discordant QFT +/TST−
Sex	Female		1	1	1
	Male		0.7 (0.06–8.04)	3.82 (0.59–24.73)	2.22 (0.7–7.14)
Age (yrs)			1.05 (0.92–1.19)	0.88 (0.75–1.04)	1.02 (0.96–1.08)
Birth in a low/moderate TB incidence country			1	1	1
Birth in a country with high TB incidence			1.26 (0.06–28.0)	2.48 (0.13–46.04)	2.3 (0.33–16.23)
Job Category	Non-clinical staff		1	1	1
	Doctors		22.54 (0.3–1683.1)	4.18 (0.16–110.99)	1.24 (0.19–7.96)
	Nurses		3.32 (0.1–214.8)	0.96 (0.08–11.92)	0.52 (0.09–2.90)
	Clinical staff		2.18 (0.1–58.1))	1.53 (0.15–15.25)	0.59 (0.14–2.66)
	Laboratory staff		-	7.96 (0.55–115.46)	-
Non-Occupational TB Exposure	No reported exposure		1	1	1
	Self-reported TB exposure		-	**28.14 (3.08–257.03)**	0.70 (0.08–6.59)
Total years worked in health care (yrs)			0.93 (0.79–1.10)	**1.23 (1.06–1.42)**	1.00 (0.94–1.08)
Travel outside Canada >1 month	No		1	1	1
	Yes		1.03 (0.09–11.1)	4.13 (0.76–22.55)	1.35 (0.43–4.24)
Worked as a HCW in a foreign country	No		1	1	1
	Yes		**30.0 (1.34–669.8)**	0.114 (0.01–2.35)	4.84 (0.87–26.89)
Self-reported occupational contact with active TB	No		1	1	1
	Yes		0.84 (0.32–2.2)	0.80 (0.42–1.54)	0.96 (0.64–1.44)
Known occupational exposure to unprotected patient with active TB	No		1	1	1
	Yes		1.13 (0.51–2.49)	1.54 (0.90–2.65)	0.94 (0.66–1.35)
No BCG vaccination			1	1	1
BCG vaccination at birth/timing unknown			3.58 (0.16–78.18)	**19.24 (1.29–287.21)**	0.84 (0.23–3.1)
BCG vaccination post-infancy or multiple vaccinations			**36.32 (1.73–761.31)**	**63.02 (3.71–1069.35)**	1.18 (0.21–6.76)
Canadian born			1	1	1
Foreign-born			10.29(0.29–361.25)	0.34 (0.03–4.57)	1.35 (0.23–7.89)
Age at immigration to Canada (yrs)			0.91 (0.78–1.07)	**1.15 (1.03–1.3)**	0.97 (0.89–1.05)
Prior TST	No prior TST or not read		1	1	1
	Prior negative TST		**0.02 (0.001–0.57)**	**0.08 (0.01–0.60)**	2.46 (0.26–23.38)

No variables were statistically significantly associated with QFT+/TST− discordance; possibly due to the very small sample size (n = 17). Several variables were associated with QFT−/TST+ discordance, including non-occupational TB exposure (OR = 28.14 96%CI: 3.08–257.03), BCG vaccination at birth/timing unknown (OR = 19.24, 95%CI:1.29–287.21) BCG vaccination after birth and/or multiple vaccinations (OR = 63.02, 95%CI: 3.71–1069.35), Total years spent working in health care (OR = 1.23, 95%CI: 1.06–1.42), age at immigration (per year increase among foreign-born), (OR = 1.15, 95%CI:1.03–1.3) and finally inverse protective effect of prior negative TST (OR = 0.08, 95%CI: 0.01–0.60).

## Discussion

Results from this cohort of Canadian HCWs suggest that the prevalence of positivity for LTBI, as measured by either the TST or the QFT, in this population, is low. Furthermore, many HCWs had discordant test results, where the prognosis and treatment recommendations remain unclear.

Infrequent test positivity (using either test) and weak concordance, may suggest that the noise level (or non-specific responses) from these tests in a low TB incidence setting may be such that the true signal (ie: immunological responses indicating LTBI) is difficult to distinguish. Non-specific variation has been shown to occur in both tests, and despite being health care workers, this cohort remains a low risk population [11], [19]. One alternative but not mutually exclusive hypothesis suggests these tests may be measuring independent immunological processes. Unfortunately, these hypotheses are difficult to test in the absence of a gold standard for LTBI.

Unlike many IGRA studies in HCWs, QFT did not estimate a lower positivity rate overall compared with the TST in this cohort [18]. Comparable positivity rates have been reported from HCW studies in the United States and Italy, and may be more frequent in cohorts with low BCG vaccination [23], [24]. The nature and profile of this particular HCW cohort is an important factor; MUHC HCWs with prior positive TST results do not undergo routine screening, and were therefore excluded. This leads to an underestimation of the overall prevalence of positive TST results among MUHC HCWs undergoing regular screening. When restricted to those HCWs who were TST naive, we found a positivity rate two-fold higher using the TST compared with QFT (9.8% vs. 4.9%). Although this did not reach statistical significance, it is consistent with earlier studies from low TB incidence countries [18]. Similar results have been reported in other North American HCW cohorts, including a conference abstract by Dorman et al., where they reported on a large multi-center study including 1,313 HCWs from the United States [25]. Both IGRAs: the QFT and the TSPOT.TB test were performed, as well as the TST, and positivity rates were 5.3%, 6.9% and 6.8% respectively. Highlighting both the low overall positivity and comparable rates estimated by the IGRAs and the TST among low risk populations.

TST+/QFT− discordant results are often attributed to BCG vaccination. In this cohort, the low level of BCG vaccination (36.1%) may lead to more comparable positivity rates between the two tests. The effect of BCG was evident when restricted to vaccinated HCWs, the TST rate increased by two fold to 12.9% while the QFT rate saw a smaller increase to 9.3%. Rates for both types of discordance remained high, and while TST+/QFT− discordance was associated with non-occupational exposure, years worked in health care, and BCG vaccination, TST−/QFT+ discordance could not be explained by any of the variables we assessed.

Results from the multivariable analysis assessing the association between LTBI risk factors and TST positivity were consistent with previous studies [18]. Given the non-specific nature of the TST, and its known cross-reactivity with BCG vaccinated persons, we are not surprised by the strong association with BCG when vaccination was performed later in life and/or repeated. Similarly, ‘total years worked in health care’ has been shown in several studies in HCWs to be consistently associated with TST positivity [26].

The association of positive TST results with self-reported non-occupational exposure was unexpected in our low TB incidence setting. However, this effect was strong and consistent across analyses, indicating non-occupational exposure to TB may continue to play an important role. Conversely, known occupational exposure to TB was not significantly associated with positive results for either test. It may be that most potential hospital source cases were appropriately diagnosed and isolated, effectively reducing nosocomial transmission. Non-occupational TB exposure may also imply longer duration plus lack of proper ventilation and infection control measures, resulting in a higher likelihood of transmission. Finally, known occupational exposures may be less likely to result in transmission than are undetected hospital exposures, which neither participants nor Occupational Health & Safety can report. QFT positivity was associated significantly with just one variable: having worked as a HCW in a foreign country, which could be a proxy for high risk exposure to TB. This variable was also significantly associated with concordant positive results, suggesting these cases may be true LTBI and not false positives.

It has been hypothesized that the TST is more likely to remain positive over time compared with the QFT, which may be a more dynamic test and has been shown to have high rates of reversions upon repeat testing [27]. It is therefore possible that TST positivity tends to be associated with cumulative TB exposure, while QFT positivity may be more linked to acute exposure, and hence less likely to persist. While it is not possible to test this hypothesis using the cross-sectional data presented here, we plan to investigate this further using future longitudinal data from this cohort.

Many have suggested alternative cut-offs for the QFT might improve reproducibility, reduce subsequent reversions and improve concordance with the TST [19], [28]. We evaluated two alternative cut-offs for the QFT test. Using the most stringent cut-off we found lower overall rates of discordance, noticeably a twofold decrease in TST−/QFT+ discordance compared with TST+/QFT− discordance. This result more closely reflects results from other studies which have consistently identified TST+/QFT− as the most frequent type of reported discordance [18].

### Strengths & Limitations

Our study was conducted in a low TB incidence setting, where TB exposure can be more accurately captured. We attempted to capture all potential exposures, both in the non-occupational and in the occupational setting; unprotected exposures to active TB patients were confirmed through occupational health and safety records. It is possible that we have not captured all occupational exposures, while TB exposure is a relatively rare event at the MUHC, and is well documented in Occupational Health and Safety charts, we cannot exclude the possibility that there were unrecognized exposures for which the HCW cannot report nor would there be any note of such an exposure in their OHS charts.

Recent work has shown that IGRAs have a certain within-subject (intra-assay) variability that can be caused by a range of factors, including: variation in sample collection and processing, improper incubation and storage, as well as laboratory factors or host biology [19], [29]. To address these concerns we attempted to reduce variability and further standardized sample processing and incubation times. Periodically, the assay was repeated on the same blood to assess reproducibility of results. However, we still cannot eliminate the possibility that some of the QFT results were false positives.

One significant limitation for all studies investigating diagnostic tests for LTBI is the lack of gold standard. While studies have looked at rates of disease progression in HCWs, this is not feasible in our low incidence setting where LTBI treatment is widely used [30].

### Conclusions

Upon one time testing of a cohort of Canadian HCWs, we found test positivity as measured by TST or QFT was low. Of concern is the high frequency of unexplainable test discordance, namely: TST−/QFT+ subjects, and the lack of association of positive tests (especially QFT) with clear-cut recent TB exposure. In settings with low TB incidence, these dynamic tests may result in such high levels of non-specific results compared with the true underlying prevalence of LTBI that more precise tests may be required to correctly identify TB. Without a clear understanding of what underlying processes each test is measuring (ie: acute, dynamic, static), and the long-term prognosis of HCWs with discordant test results, it remains unclear how to implement QFT tests into current HCW screening programs, and what additional value they may offer over the TST.
